# Spallation and particles infusion into the extracorporeal circuit during CRRT: a preventable phenomenon

**DOI:** 10.1038/s41598-024-59245-7

**Published:** 2024-04-20

**Authors:** Maria Cristina Ruffa, Giacomo Bignante, Vittorio Bocciero, Sergio Fabbri, Dario Degl’Innocenti, Valentina Cauda, Gianluca Villa

**Affiliations:** 1 Department of Applied Science and Technology, Politecnico di Torino, Turin, Italy; 2 Department of Health Sciences, Section of Anesthesiology, Intensive Care and Pain Medicine, University of Florence, Florence, Italy; 3 Department of Anesthesia and Intensive Care, Section of Oncological Anesthesia and Intensive Care, Careggi Hospital, Florence, Italy

**Keywords:** In-line filtration, Plastic bags, Replacement, CRRT solutions, Membrane fouling, Nephrology, Risk factors, Biomedical engineering, Characterization and analytical techniques, Kidney diseases, Acute kidney injury, Chronic kidney disease, Paediatric kidney disease

## Abstract

Patients in intensive care are exposed to the risk of microparticle infusion via extracorporeal lines and the resulting complications. A possible source of microparticle release could be the extracorporeal circuit used in blood purification techniques, such as continuous renal replacement therapy (CRRT). Disposable components of CRRT circuits, such as replacement bags and circuit tubing, might release microparticles such as salt crystals produced by precipitation in replacement bags and plastic microparticles produced by spallation. In-line filtration has proven effective in retaining microparticles both in in-vitro and in-vivo studies. In our study, we performed an in-vitro model of CRRT-treatment with the aim of detecting the microparticles produced and released into the circuit by means of a qualitative and quantitative analysis, after sampling the replacement and patient lines straddling a series of in-line filters. Working pressures and flows were monitored during the experiment. This study showed that microparticles are indeed produced and released into the CRRT circuit. The inclusion of in-line filters in the replacement lines allows to reduce the burden of microparticles infused into the bloodstream during extracorporeal treatments, reducing the concentration of microparticles from 14 mg/mL pre in-line filter to 11 mg/mL post in-line filter. Particle infusion and related damage must be counted among the pathophysiological mechanisms supporting iatrogenic damage due to artificial cross-talk between organs during CRRT applied to critically ill patients. This damage can be reduced by using in-line filters in the extracorporeal circuit.

## Introduction

The release of inorganic particles into the patient’s bloodstream is a rising concern, particularly in the critical care setting, where patients are exposed to a high volume of intravenous infusions. Up to one million particles can be infused daily in this setting due to drug and salt precipitation, drugs aggregates, contamination of infused solutions from glass ampoules, as well as micro and nanoplastics^1,2^ mainly deriving from infusion sets and infusion bags (e.g., plastic/glass particulate, microscopic cellulose fibres, micro- and macro-rubber particles). Post-mortem studies have demonstrated the presence of plastic and glass particles in the vessels and lungs of critically ill patients, resulting in mechanical blockage of the microcirculation, local inflammation, and tissue granuloma^2,3^. Several studies have demonstrated the capability of intravenously infused particles to induce endothelial damage, inflammation, and immune system activation^4,5^. Particle retention positively affects surgical and critically ill patients, especially when treated with high amounts of intravenous fluids and drugs. In-line filters are disposable products placed inside the intravenous line to retain particles and prevent unintentional administration to the patient through the vascular access device. Villa et al. demonstrated that in-line filters significantly reduce endothelial damage in surgical patients^4,5^. Furthermore, Sasse et al. observed a significant reduction in the development of systemic inflammatory disorders and multiple organ dysfunction in a cohort of intensive care unit (ICU) patients when in-line filtration was applied to prevent the infusion of intravenous particles during the ICU stay^1^. Continuous renal replacement therapy (CRRT) is a widely adopted extracorporeal blood purification (EBP) therapy in the intensive care unit (ICU), where up to 10% of patients with acute kidney injury (AKI) require CRRT during their ICU stay^6^. As with other EBPs, immunomodulation may be a therapeutic target in critically ill patients with AKI and systemic inflammation (e.g., during sepsis-associated AKI). To be delivered, CRRT requires plastic lines and bags that could release particles into the bloodstream, potentially clogging the haemodiafilter or reaching the patient resulting in endothelial damage and systemic inflammation. Breakage of the thermal seal and mechanical agitation of the replacement bags could cause the release of plastic materials into the replacement fluids and thus into the extracorporeal circuit. Furthermore, repeated compression of peristaltic pumps on circuit lines induces mechanical stress, fragmentation, and detachment of tube particles. This phenomenon is called spallation^7^. Several studies have correlated spallation-induced particles with different pathological phenomena: increased production of IL-1 by macrophages^8^, local and systemic endothelial activation^9^, and patient’s organ damage^10^, particularly among patients undergoing haemodialysis^8^. Considering the high volume of replacement fluids used during CRRT and the considerable spallation occurring during extracorporeal treatments performed for several hours (often days) in the ICU, it can be assumed that a significant number of particles are delivered to patients during EBPs. This in-vitro study aims to verify the presence of particles in the extracorporeal circuit and the effectiveness of in-line filtration in retaining them.

## Materials and methods

**Figure 1:**
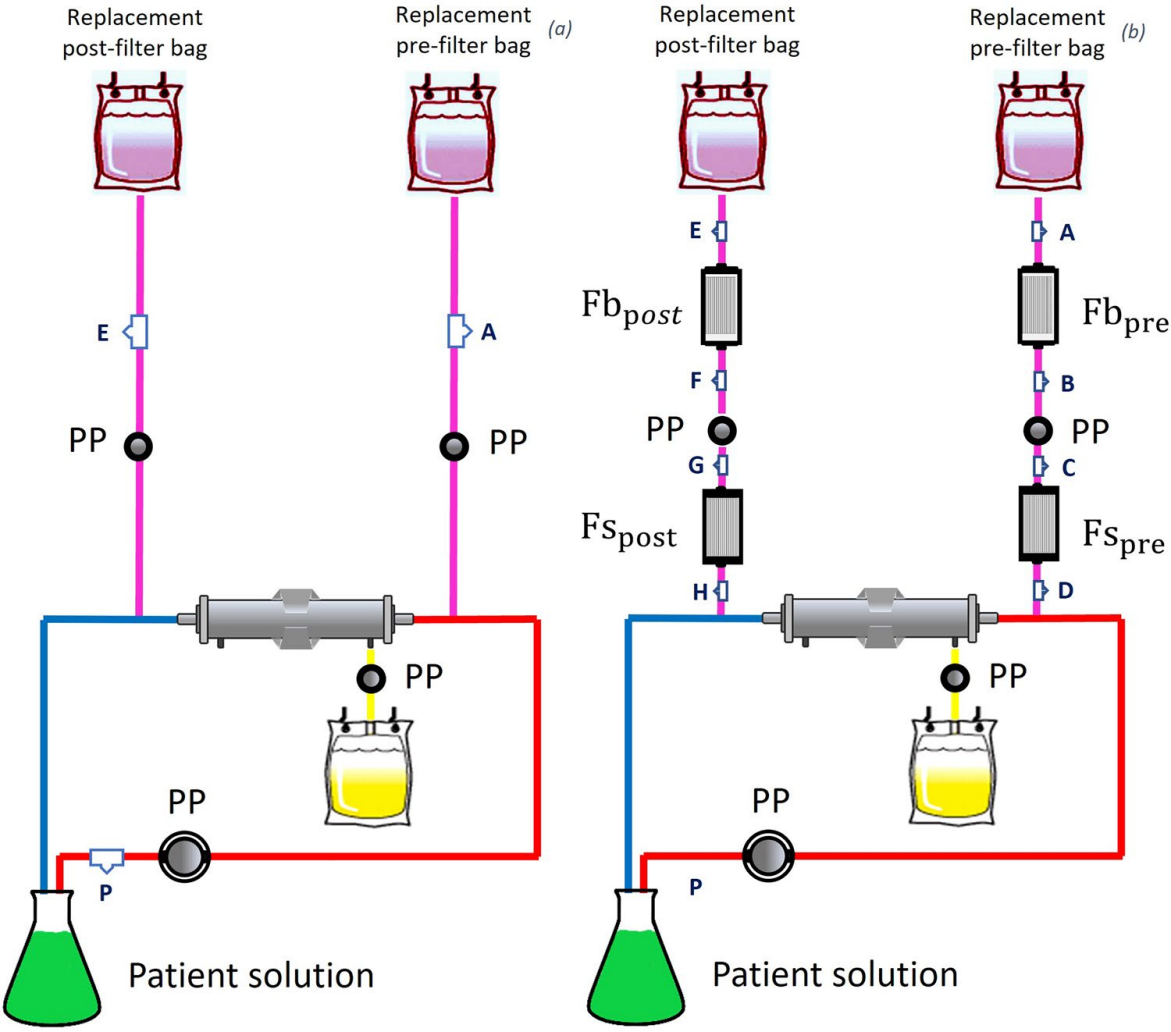
Experimental sets: “control phase” (**a**) and “filtering phase” (**b**). Peristaltic pumps: PP. Circuit sampling points: A, B, C, D, E, F, G, H, P. In-line filters: ${{\text{Fb}}}_{{\text{pre}}}$ and ${{\text{Fb}}}_{{\text{post}}}$ (in-line filters for replacement bags pre and post replacement lines), ${{\text{Fs}}}_{{\text{pre}}}$ and ${{\text{Fs}}}_{{\text{post}}}$ (in-line filters for spallation pre and post replacement lines).

In vitro experiments simulating continuous veno-venous haemofiltration (CVVH) were performed at the “Laboratory of Extracorporeal Blood Purification Therapy’’ of the Department of Health Sciences, Section of Anaesthesiology, Intensive Care and Pain Medicine, University of Florence. CVVH was carried out using Prismaflex® (Baxter, Deerfield, IL, USA) with an M100 (Baxter, Deerfield, IL, USA) extracorporeal circuit kit and was performed for 5 h in pre- and post-dilution (PrismaSol® 4mmol/L, Baxter). Prismaflex system automatically conducts standard occlusivity test of peristaltic pumps. The M100 circuit is made by tubes in PVC and haemodiafilter membrane in acrylonitrile and sodium methallyl sulfonate copolymer. The effluent dose was set at 28 mL/kg/h for an ideal 70 kg patient with 30% hematocrit and no net ultrafiltration was applied. The treatments were conducted with 3 L of 0.9% saline solution, i.e., the “patient solution” at room temperature. Thus, anticoagulation was not necessary. During the treatments, transmembrane pressure (TMP) was continuously recorded by the CRRT machine, using standard formula. Two equations describing the TMP trend for each of the study groups were calculated to show their difference in slope. Two different non-consequential experimental sets were repeated five times (N = 5). In the first setting, i.e., the “control phase”, the CRRT treatment was performed according to the methods listed above. In the second one, i.e. the “filtering phase”, four in-line particle retention filters ($11 {{\text{ cm}}}^{2}$ positively charged membrane with pores size equal to 0.2 µm; ELD96LLCE; Pall, Dreieich, Germany) were applied in the replacement lines. Two were placed between the replacement bags and the replacement peristaltic pumps (${{\text{Fb}}}_{{\text{pre}}}$ and ${{\text{Fb}}}_{{\text{post}}}$), and the other two were placed between the peristaltic pumps and the haemodiafilter (${{\text{Fs}}}_{{\text{pre}}}$ and ${{\text{Fs}}}_{{\text{post}}}$). ${{\text{Fb}}}_{{\text{pre}}}$ and ${{\text{Fb}}}_{{\text{post}}}$ should theoretically depurate the replacement solution of particles from the plastic bags, while ${{\text{Fs}}}_{{\text{pre}}}$ and ${{\text{Fs}}}_{{\text{post}}}$ should retain particles produced by the spallation due to peristaltic pumps driving the replacement fluids (Fig. 1).

During the 5-h treatment period, fluid samples (5 mL) were taken from different access points in the circuit. Specifically, in the “control phase”, samples were taken at the beginning and at the end of the treatment from the “patient solution” (P circuit sampling points) and from the replacement lines (E and A circuit sampling points) to analyze the overtime changes of particles released within the circuit. In the “filtering phase”, on the other hand, samples were collected from the “patient solution” (P circuit sampling points) and along the replacement lines at the initiation and interruption of CRRT, before (A, C, E, G circuit sampling points) and after (B, D, F, H circuit sampling points) the in-line filters for particle retention, in order to demonstrate the presence of particles due to plastic bag replacement and/or spallation and the effectiveness of in-line filters in their retention.

The samples obtained from “patient solution” were analyzed using a qualitative method through optical microscopy. The samples were centrifuged at 14,000×*g* for 30 min at 20 °C. After that, the pellet was resuspended in 100 μL of microfiltered double distilled water and recentrifugated. The washed pellet was resuspended in 100 μL and then 20 μL of solution for each sample were deposited a glass slide, coated by a cover glass slip, and was analyzed under an optical inverted microscope (Ti-E Nikon) operating in bright field at × 40 magnification. For each sample, 10 representative images were acquired. Instead, for the samples obtained along the replacement lines, both qualitative and quantitative analytical methods were used to evaluate the particle count. The qualitative analysis was performed again by optical microscope, using the same procedure as described above. The quantitative method used the weight of dry residue. In details, 5 mL glass tubes (mean and standard deviation of the empty tube weight: 6.2714 g ± 0.0003) were filled with 4 mL of each sample solution and allowed to dry in an oven at 60 °C. Once dried, using the data of the empty and dry weight of the tubes, the dry residue weight of the solution was calculated by subtracting the weight of the tube with dry residue from the weight of the dried but empty tubes. Quantitative variables are reported as median/interquartile range according on the distribution of the data. Additionally, Field Emission Scanning Electron Microscope and Energy Dispersive X-Ray Spectroscopy (FESEM-EDX) were employed to analyze the morphology and chemical composition of the particles detected in the dried samples. 40 μL of solution for each sample was deposited on a silicon wafer and this was analysed by FESEM (Merlin from Carl Zeiss) operating at 10 kV and coupled with EDX (Oxford Instrument, equipped with Inca software).

### Statement of ethics

An ethics statement was not required for this study type, no human or animal subjects or materials were used.

## Results

**Figure 2:**
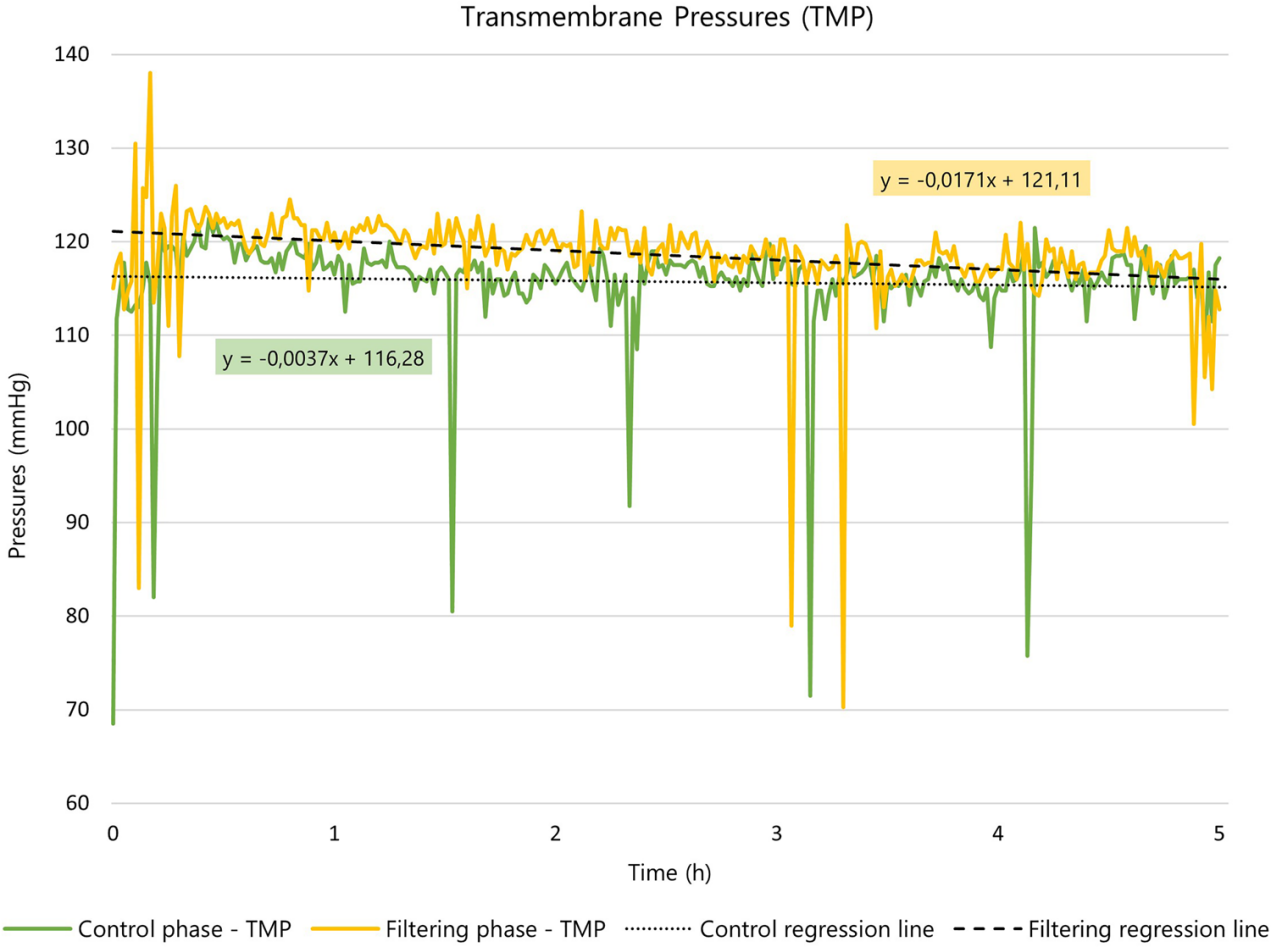
Transmembrane pressures (TMP) in “control phase” and “filtering phase”.

The experiments were carried out with an effective treatment time of 303 min [295–309 min], in accordance with the set time frame (5 h). There were no alarms during the treatments and the transmembrane pressure (TMP) was maintained with an average of 115 mmHg in “control phase” and 118 mmHg during the “filtering phase” (Fig. 2). Comparison of the equations parameterising the two trend lines, shown in Fig. 2, highlight the slopes of the two TMP signals.

Specifically, the slopes of the two curves, as shown in the equations in Fig. 2 are approximately zero (− 0.0037 for control and − 0.0171 for filtering) for both configurations. Therefore, for both the control and filtering phases, the slopes of the curves are similar.

**Figure 3:**
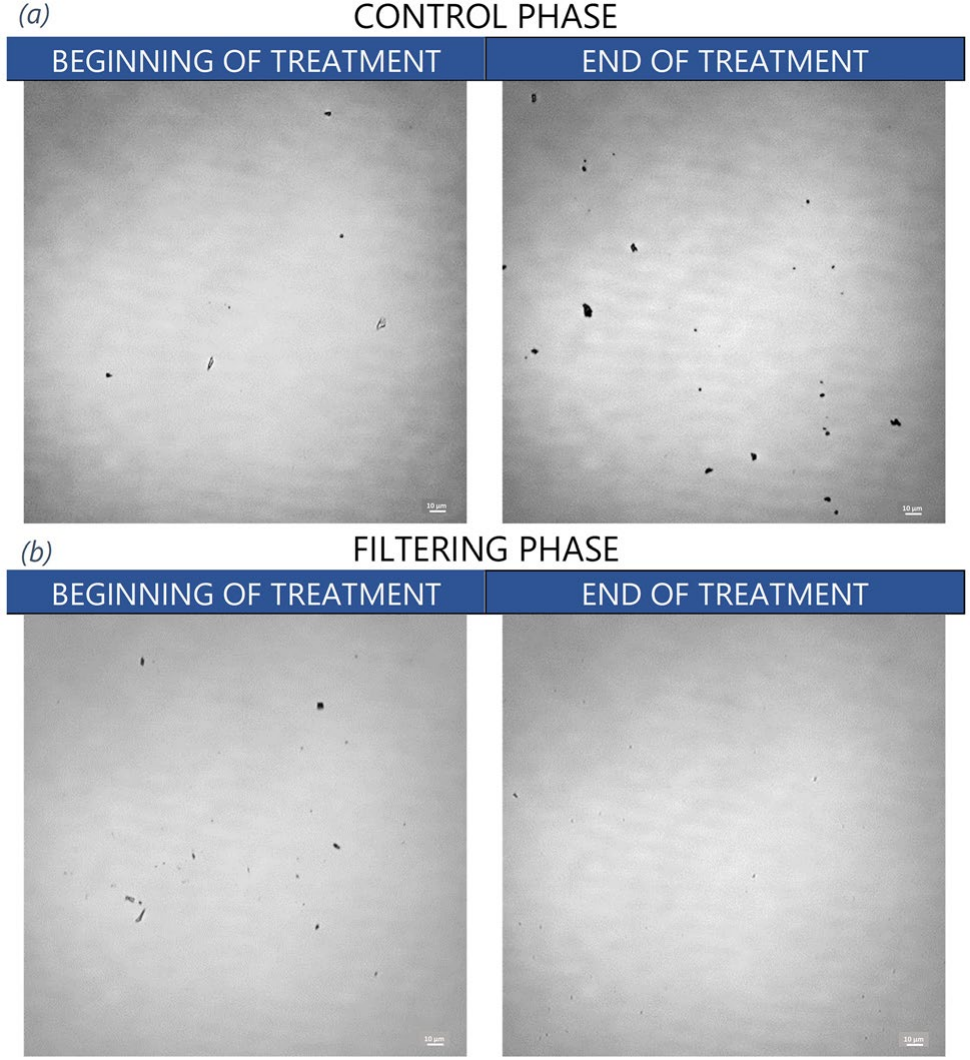
Wide-field optical microscope images of the “patient line” solutions (P circuit sampling point) in “control phase” (**a**) and in “filtering phase” (**b**) at the CRRT initiation and interruption. The scale bars are of 10 μm.

Wide-field optical microscope images highlight the particles, mainly micrometer-sized, detected in the “patient solution” at the beginning and interruption of the treatment either with or without the use of in-line filtration. The Fig. 3 shows the different qualitative behaviour between “control phase” and “filtering phase” in the “patient solution”. The average particle diameter in both “control” and “filtering” phases at the beginning of the treatment is about 2 μm, while at the end of the treatment it is of 4 μm in the “control phase” and 0.6 μm in the “filtering phase”.

Additional images acquired by wide-field optical microscopy are in the Supplementary Information (Figs. S1–S5).

**Figure 4:**
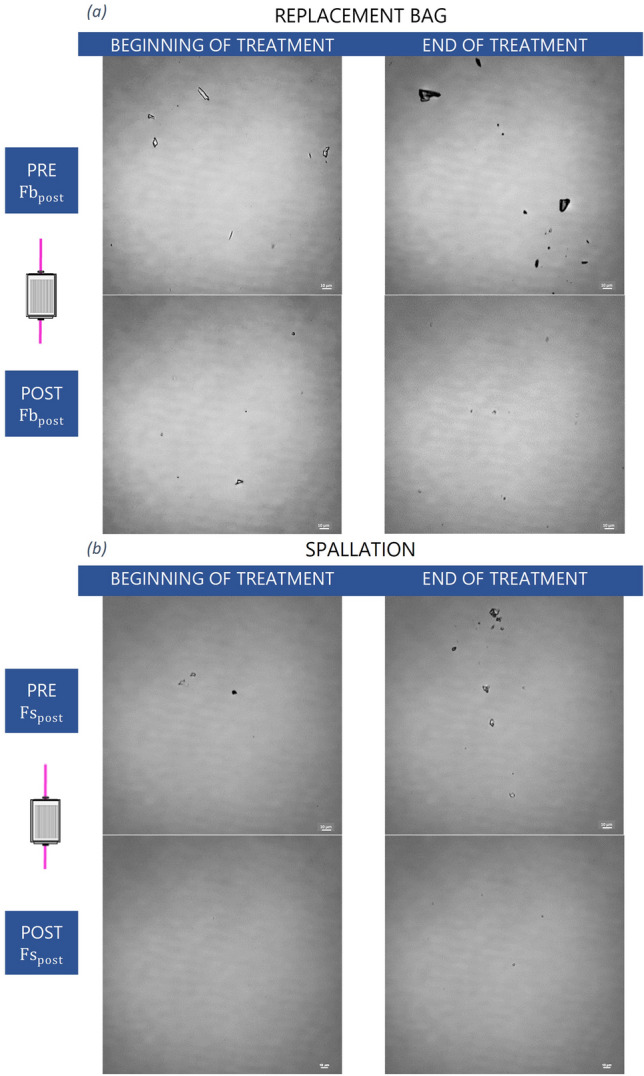
Wide-field optical microscope images of solutions sampled from the post-filter replacement line. Representation of treatment at the beginning and interruption of CRRT downstream of the replacement bag (**a**) (E, F circuit sampling points) and downstream of the peristaltic pump (**b**) (G, H circuit sampling points). The scale bars are of 10 μm.

In the “filtering” phase, the wide field optical microscope also analyzed the presence of the particles in the replacement lines. Figure 4 shows the representative images of a single replacement line, while the behavior of the two lines is similar. Particles found at the beginning and interruption of CRRT, straddling the ${{\text{Fb}}}_{{\text{pre}}}$, ${{\text{Fb}}}_{{\text{post}}}$, ${{\text{Fs}}}_{{\text{pre}}}$ and ${{\text{Fs}}}_{{\text{post}}}$ in-line filters, were highlighted.

Additional images acquired by wide-field optical microscopy are in the Supplementary Information (Figs. S6–S13).

**Figure 5:**
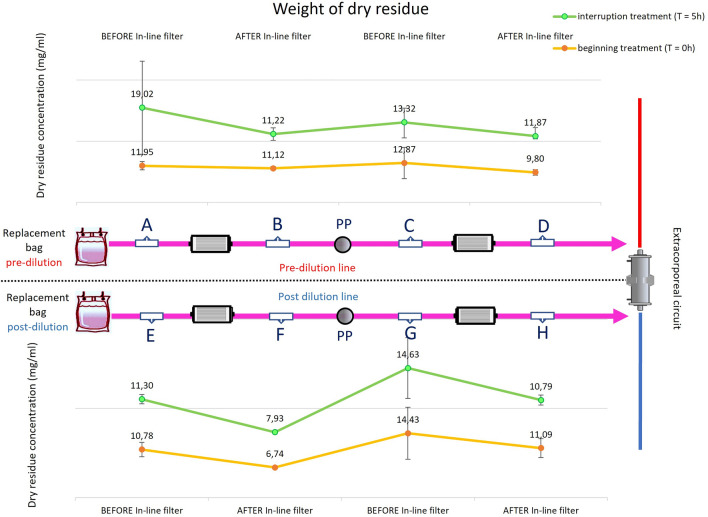
Medians and interquartile range of the dry residue concentrations (N = 5) obtained from the replacement lines (pre-haemodifilter, top panel- and post-haemodiafilter, bottom panel) evaluated straddling the in-line filters (${{\text{Fb}}}_{{\text{pre}}}$, ${{\text{Fb}}}_{{\text{post}}}$, ${{\text{Fs}}}_{{\text{pre}}}$ and ${{\text{Fs}}}_{{\text{post}}}$) at the beginning (yellow lines) and end (green lines) of the treatment.

Quantitatively, particle loading was evaluated in the two replacement lines, pre-dilution, and post-dilution. Assuming that the amount of salts contained within each replacement bag, and therefore of each sample, is the same, the weight of microplastic particles present in each sample, and thus their concentration, can be assessed. Figure 5 shows the trend of the dry residue, in terms of concentration, i.e., residue weight normalized by the dried solution volume, taken straddling the ${{\text{Fb}}}_{{\text{pre}}}$, ${{\text{Fb}}}_{{\text{post}}}$, ${{\text{Fs}}}_{{\text{pre}}}$ and ${{\text{Fs}}}_{{\text{post}}}$ in-line filters in two replacement lines at the beginning and end of CRRT treatment.

Additional images is given in the Supplementary Information. One showing a quantitative analysis of particle concentration (n.particles/μL) (Fig. S16) and other one the amount of residue in the patient solution, P points (Fig. S17).

**Figure 6:**
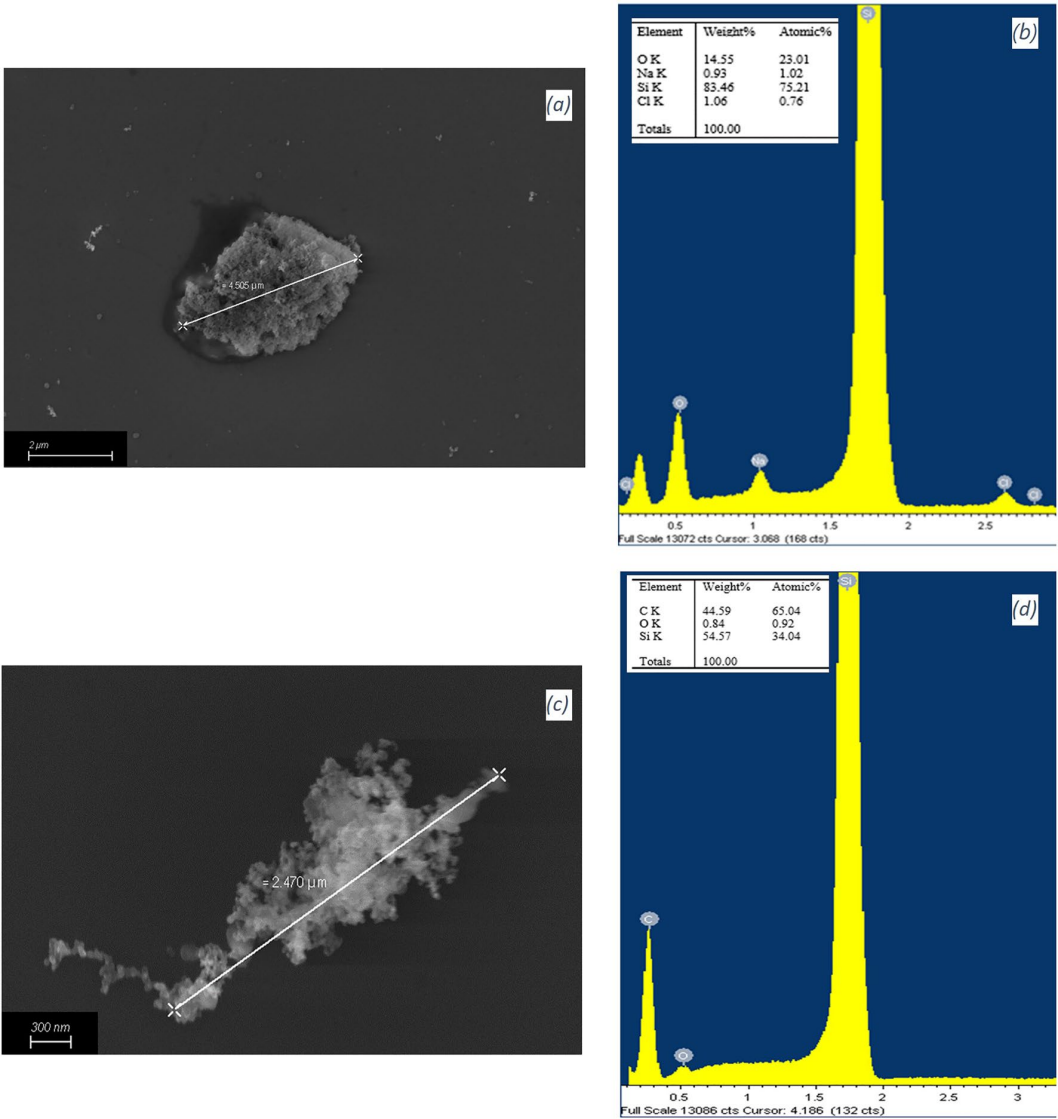
Examples of salt (**a**) and microplastic particle (**c**) detected within samples acquired by FESEM at 20.00 KX and 100.00 KX, respectively. The spectra detected by EDX analysis show the chemical composition of the salt particles (**b**) (NaCl) and of the microplastic particles (**d**) (C–O). The Si detected derives from the wafer substrate.

Finally, a chemical and morphological analysis of the microparticles contained in the different samples was performed using the FESEM-EDX method. Straddling the ${{\text{Fb}}}_{{\text{pre}}}$, ${{\text{Fb}}}_{{\text{post}}}$ in-line filters are predominantly salt particles contained in the replacement bags, while straddling the ${{\text{Fs}}}_{{\text{pre}}}$ and ${{\text{Fs}}}_{{\text{post}}}$ in-line filters are predominantly micro-sized plastic particles resulting from the wear of the circuit tubes that are continuously compressed by peristaltic pumps (Fig. 6). The salts consist mainly of elements such as sodium and chlorine. The microplastic particles, on the other hand, are composed of carbon and oxygen and morphologically are an aggregate of nanosized particles. Additional images acquired by FESEM-EDX methods are in the supplementary information (Figs. S14, S15).

## Discussion

This in-vitro study shows that microparticles are actually produced into the circuit during CRRT and released to patients. The experiment was carried out at room temperature to reproduce the real-life conditions of use. These particles mainly consist of salt crystals produced by precipitation in the replacement bags and plastic substances produced by spallation processes. The inclusion of in-line filters in replacement lines reduces the number of particles released into the bloodstream and loaded onto patients during extracorporeal treatments. To our knowledge, this is the first study to explore the formation of particles, their loading and retention during CRRT. Most patients undergoing CRRT already have severe hyperinflammation and EBPs are mainly indicated for immunomodulation. This article demonstrates that a significant particle load derives from CRRT. Considering the literature describing the biological effects of circulating particles in critically ill patients, it can be postulated that CRRT itself may paradoxically worsen systemic inflammation and organ dysfunction due to particle release. Notably, particles have been identified in the literature in the lungs and kidneys of critically ill patients treated with high volumes of intravenous fluids in the ICU. The findings of this article may suggest that particle infusion and related damage should be counted among the pathophysiological mechanisms sustaining iatrogenic damage due to artificial cross-talk between organs during EBP applied to critically ill patients. The increase in particles demonstrated in the patient’s solution in the control phase certainly has a multiple origin, e.g., from the atmosphere, from the glass flask, etc. The presence of particles observed in the replacement line in both experimental phases shows that at least some of them derive from salt precipitates in the replacement bags and plastic fragmentation due to spallation. Interestingly, microparticles were present from the beginning of treatment but were not generated in a linear fashion; they reached maximum levels at the end of treatment (5 h). The observed particulate matter is the same at the beginning of treatment for both experimental sets (same dry residue concentration at sampling points A and E at the beginning of treatment in filter phase and control phase). In the «control phase», the particulate matter that is generated by bags and tubes ends up directly in the circuit and thus theoretically in the patients blood, while in the «filter phase» the amount of particulate matter is abated by the presence of in-line filters. The explanation for the increase in particulate matter over time in the «control phase» could be the mechanical derangement of the components of the disposable circuit, mainly due to the compression and expansion cycles induced by the activity of the peristaltic pumps, which induce spallation of the tubes, in agreement with the results of previous in vitro studies^7,10^. Replacement bags could also play a role in the process, due to the time-dependent formation of saline precipitates, favoured by the sedimentation of a stagnant saline solution within the replacement bags. Additionally, the broken thermowelding and the plastic material of the bag could be the main cause of microparticles within the extracorporeal circuit at the beginning of the treatment. In this study, inconclusive results were obtained in exploring the effect that particles may have in inducing membrane pore occlusion. In particular, no signs of membrane clogging were demonstrated in either the control or filtration phase. No specific alarms were detected, nor was an increase in TMP demonstrated in the control phase. These results may suggest that particles are not able to significantly occlude the membrane pore (probably according to their relative dimensions). Nonetheless, the limited effective treatment time used for the experiments and, above all, the use of crystalloid solutions as “patient solutions” may have underestimated these expected effects. The direct contact between the patient’s blood and the particles retained in the haemodiafilter membrane may increase the activation of clotting locally, potentially inducing membrane fouling. Unfortunately, we were not able to explore this effect in-vivo. Beyond the limited effective treatment time and the adoption of crystalloid solution instead of blood products to conduct the experiments, other limitations can be recognized in this study. Firstly, we are unable to fully demonstrate the origin of the particles observed in the patient’s solution in the control phase. In particular, we are unable to exclude spallation from the blood pump, which could contribute to the particle load. Above all, we are unable to quantify the significance of this contribution. Additionally although particulate matter collected by in-line filters has been photographed by SEM in other studies, we are not able with our capabilities to break it down without creating new plastic particles. Secondly, providing a sample (control) that contains only and exclusively microparticles is unfortunately not possible. These, in fact, are generated by dynamic phenomena, such as rupture of the bag heat seal or crushing of the tubing by the peristaltic pump. These dynamic phenomena mix the microparticles formed with the salt solutions flowing within the extracorporeal circuit. Therefore, the collected samples will consist of both salts and microparticles. Viceversa, it is also not possible to collect the solution only with salts without microparticles (i.e. as a control solution), as the bag is made by two separated compartments with different salts which are mixed immediately before the CRRT process starts and their mixing undoubtedly implies the generation of microparticles, i.e. due to the rupture of the bag heat seal. Thirdly, we have applied CVVH as an experimental treatment to reduce the variables influencing the results, whereas it is known that continuous veno-venous haemodiafiltration is the most widely used treatment at the patient’s bedside. Exploration of clinical benefit is beyond the scope of this study, which is intended to be only a proof of concept. Infact we did not calculate sample populations and preferred not to express inferential statistics but only descriptive statistics. Therefore, we cannot quantify the possible particle load from the dialysate bags and spallation by the related dialytic pump.

## Conclusion

This study shows the long-standing issue of the interaction between extracorporeal circuits and biological fluids: the risk of microparticle release is very high during CRRT, and could cause a time-dependent burden of microparticle administration to the patient. Experiments also demonstrate that microparticle formation pathways involve solution stagnation in fluids bags and spallation. Interestingly, in-line filtration retain particles during treatment.

The amount and clinical consequences of particle load for patients treated with CRRT should be explored in-vivo in further studies. Likewise, the clinical benefits of including in-line filters in extracorporeal circuits should be evaluated in detail.

### Supplementary Information

Supplementary Information.

## Data Availability

Data will be made available to those researchers that will contact the authors asking for them and explaining the intention of their use. All data generated or analysed during this study are included in this article. Further enquiries can be directed to the corresponding author.
