# Supplementary material for: Spallation and particles infusion into the extracorporeal circuit during CRRT: a preventable phenomenon
Source: Sci Rep. 2024 Apr 20;14:9089. doi: 10.1038/s41598-024-59245-7 (PMC11032310; doi:10.1038/s41598-024-59245-7)
Supplement: Supplementary file 1 — Supplementary Information. [file 41598_2024_59245_MOESM1_ESM.docx]

**SUPPORTING INFORMATION**

- Wide-field optical microscope images

*Figure S1*. Wide-field optical microscope images of the “patient line” solutions (P circuit sampling point) in “control phase” at the CRRT initiation. The scale bars are of 10 μm.

*Figure S2*. Wide-field optical microscope images of the “patient line” solutions (P circuit sampling point) in “control phase” at the CRRT interruption. The scale bars are of 10 μm.

*Figure S3.* Wide-field optical microscope images of the “replacement line” solutions in “control phase” sampled by replacement pre filter bag (a) (A circuit sampling point) and replacement post filter bag (b) (E circuit sampling point) at the CRRT initiation and interruption. The scale bars are of 10 μm.

*Figure S4*. Wide-field optical microscope images of the “patient line” solutions (P circuit sampling point) in “filtering phase” at the CRRT initiation. The scale bars are of 10 μm.

*Figure S5*. Wide-field optical microscope images of the “patient line” solutions (P circuit sampling point) in “filtering phase” at the CRRT interruption. The scale bars are of 10 μm.

*Figure S6.* Wide-field optical microscope images of solutions sampled from the post-filter replacement line. Representation of treatment at the beginning of CRRT downstream of the replacement bag (E circuit sampling points). The scale bars are of 10 μm.

*Figure S7.* Wide-field optical microscope images of solutions sampled from the post-filter replacement line. Representation of treatment at the beginning of CRRT downstream of the replacement bag (F circuit sampling points). The scale bars are of 10 μm.

*Figure S8.* Wide-field optical microscope images of solutions sampled from the post-filter replacement line. Representation of treatment at the interruption of CRRT downstream of the replacement bag (E circuit sampling points). The scale bars are of 10 μm.

*Figure S9.* Wide-field optical microscope images of solutions sampled from the post-filter replacement line. Representation of treatment at the interruption of CRRT downstream of the replacement bag (F circuit sampling points). The scale bars are of 10 μm.

*Figure S10.* Wide-field optical microscope images of solutions sampled from the post-filter replacement line. Representation of treatment at the beginning of CRRT downstream of the peristaltic pump (G circuit sampling points). The scale bars are of 10 μm.

*Figure S11.* Wide-field optical microscope images of solutions sampled from the post-filter replacement line. Representation of treatment at the beginning of CRRT downstream of the peristaltic pump (H circuit sampling points). The scale bars are of 10 μm.

*Figure S12.* Wide-field optical microscope images of solutions sampled from the post-filter replacement line. Representation of treatment at the interruption of CRRT downstream of the peristaltic pump (G circuit sampling points). The scale bars are of 10 μm.

*Figure S13.* Wide-field optical microscope images of solutions sampled from the post-filter replacement line. Representation of treatment at the interruption of CRRT downstream of the peristaltic pump (H circuit sampling points). The scale bars are of 10 μm.

- FESEM-EDX


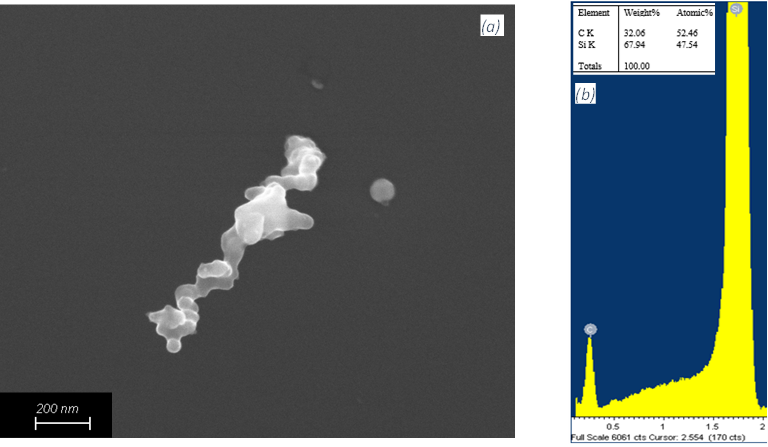

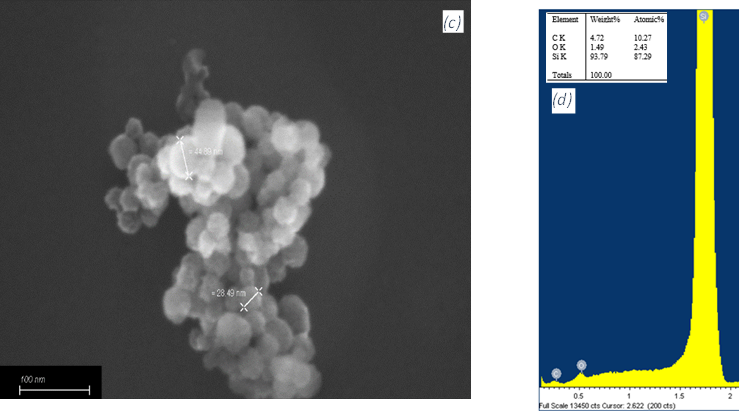


*
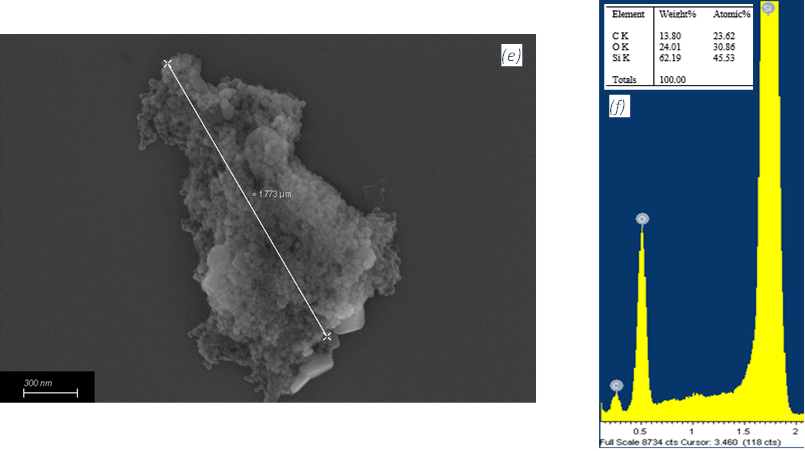
*

*Figure S14*. Examples of microplastic particle (a-c-e) detected within samples acquired by FESEM at 100.00KX and 400.00KX. The spectra detected by EDX analysis show the chemical composition of the microplastic particles (b-d-f) (C-O). The Si detected derives from the wafer substrate


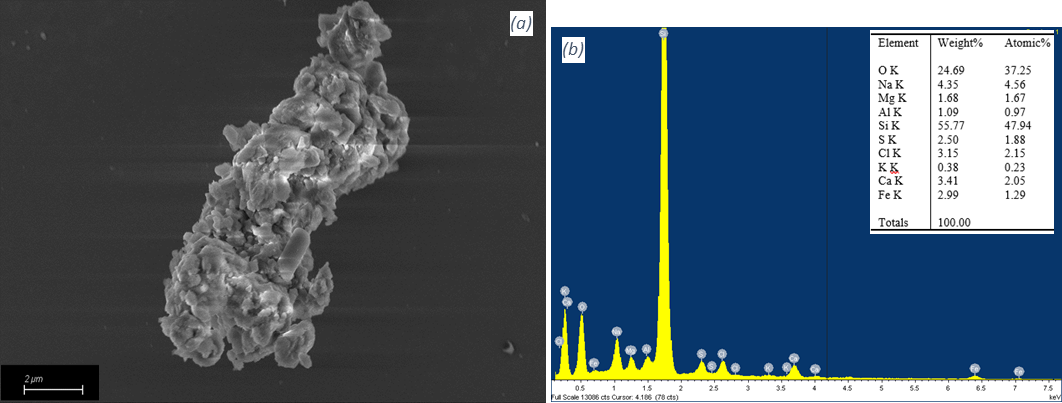


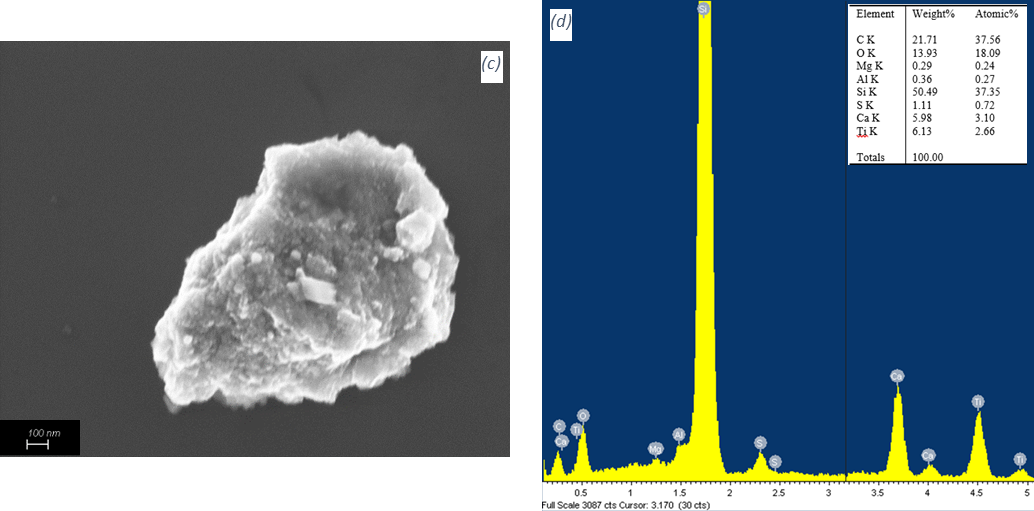


*Figure S15*. Examples of particle (a-c) detected within samples acquired by FESEM at 15.00KX and 100.00KX. The spectra detected by EDX analysis show the chemical composition of the microplastic particles (b-d) (O-Na-Mg-Al-S-Cl-K-Ca-Fe-C-Ti). The Si detected derives from the wafer substrate.

- Wide-field optical microscope images and NIS-Elements

(b)

(a)

*Figure S16*. Particle concentration as a function of the diameter for the samples collected pre and post in-line filter (blue and orange, respectively). Specifically, in the panel (a) are shown the samples taken downstream of the replacement bag while, in the panel (b) those taken downstream of the peristaltic pump.

-
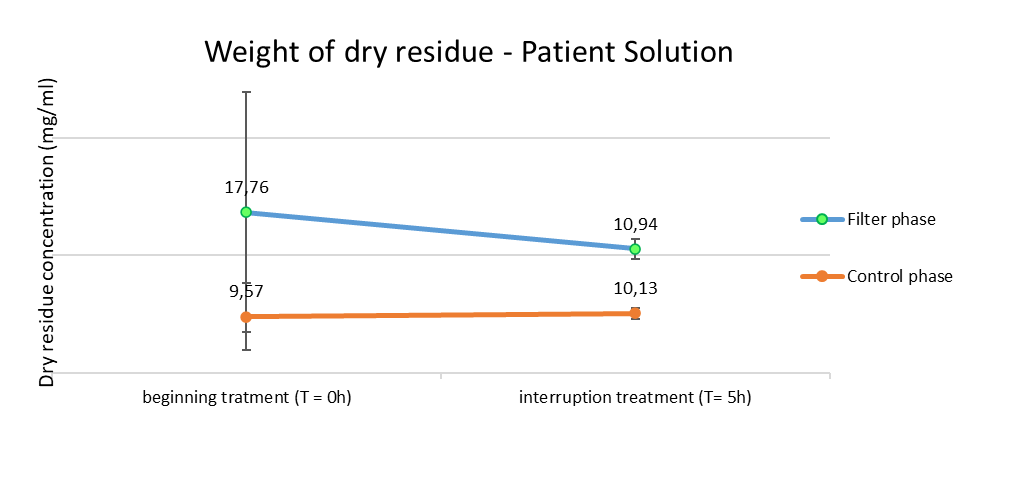
Weight of dry residue

*Figure S17*. The amount of residue at the patient points (P) with (blue line) and without (orange line) the filters.

- Occlusion setting of roller pump:

| Occlusivity test | Prismaflex system automatically conducts standard occlusivity test of peristaltic pumps. The protective system commands the control to stop all the pumps and close the return line clamp. The protective system then commands the pump to start turning until the return pressure is greater than +400mmHg. While the blood pump is turning, the protective system counts the number of rotations required to achieve this pressure using the encoder. The number of rotations must not be greater than a specific value dependent on the disposable. The protective system reads the values of the filter, effluent and return pressure sensors. After waiting an additional 10 seconds the protective system again reads the pressure values and compares them with the initial recorded values. Depending on which set is used the pressure change should be less than 8 to 18 mmHg. |
| --- | --- |
